# Biological efficacy of perpendicular type-I collagen protruded from TiO_2_-nanotubes

**DOI:** 10.1038/s41368-020-00103-3

**Published:** 2020-12-30

**Authors:** Chia-Yu Chen, David. M. Kim, Cliff Lee, John Da Silva, Shigemi Nagai, Toshiki Nojiri, Masazumi Nagai

**Affiliations:** 1 Department of Oral Medicine, Infection and Immunity, Harvard School of Dental Medicine, 188 Longwood Avenue, Boston, MA USA; 2 Department of Restorative Dentistry and Biomaterials Sciences, Harvard School of Dental Medicine, 188 Longwood Avenue, Boston, MA USA; 3 Department of Prosthodontics and Oral Implantology, School of Dental Medicine, Iwate Medical University, 19-1 Uchimaru, Morioka, Iwate Japan

**Keywords:** Dental biomaterials, Preclinical research

## Abstract

The aim of this study was to evaluate the biological efficacy of a unique perpendicular protrusion of type-I collagen (Col-I) from TiO_2_ nanotubes (NT-EPF surface). We hypothesized that the NT-EPF surface would play bifunctional roles in stimulating platelet-mediated fibroblast recruitment and anchoring fibroblast-derived Col-I to form a perpendicular collagen assembly, mimicking the connective tissue attachment around natural teeth for the long-term maintenance of dental implants. Ti surface modification was accomplished in two steps. First, TiO_2_ nanotubes (NT) array was fabricated via anodization. Diameters and depths of NTs were controlled by applied voltage and duration. Subsequently, an electrophoretic fusion (EPF) method was applied to fuse Col-I into nanotube arrays in a perpendicular fashion. Surface wettability was assessed by contact angle measurement. The bioactivity of modified TiO_2_ surfaces was evaluated in terms of NIH3T3 fibroblast attachment, platelet activation, and collagen extension. Early attachment, aggregation, and activation of platelets as well as release of platelet-related growth factors were demonstrated on NT-EPF surfaces. Platelet-mediated NIH3T3 cells migration toward NT-EPF was significantly increased and the attached cells showed a typical fibrous morphology with elongated spindle shape. A direct linkage between pseudopod-like processes of fibroblasts to NT-EPF surfaces was observed. Furthermore, the engineered EPF collagen protrusion linked with cell-derived collagen in a perpendicular fashion. Within the limitation of this in vitro study, the TiO_2_ nanotube with perpendicular Col-I surface (NT-EPF) promoted better cell attachment, induced a strong platelet activation which suggested the ability to create a more robust soft tissue seal.

## Introduction

Since the introduction of the concept of *osseointegration* by Branemark et al.,^1^ the use of endosseous dental implants for rehabilitation of missing teeth has become a routine treatment option. However, implant-related complications are frequently being reported and failures do occur. Peri-implantitis is a plaque-associated pathological condition occurring in tissues around dental implants, characterized by inflammation in the peri-implant mucosa and subsequent progressive loss of supporting bone,^2^ with an estimated prevalence of 22%, with reports up to 47%.^3^

Although plaque is the main etiologic factor in both peri-implantitis and periodontal disease, there are many differences between the two disease entities. Bone loss progresses more rapidly around dental implants compared to natural teeth, and often continues even after the etiology is removed.^4,5^ The weak soft tissue barrier around titanium implants due to the lacks of rigid epithelial and connective tissue (CT) attachments could be a likely cause.^6^ The periodontium of natural teeth is protected from bacterial invasion by the firm sealing of the junctional epithelium, attaching to the tooth surface via a basement lamina (BL).^7^ Furthermore, the perpendicular attachment of dentogingival CT fibers prevents the apical migration of the epithelium.^8,9^ In contrast, the peri-implant epithelium is clinically recognized to have poor attachment with few basement membrane facing the implant.^10^ An even greater difference is observed at the CT level whereby bundles of collagen fibers run parallel and circumferentially around the implant surface and are thus unable to resist epithelial downgrowth.^8,9^

To minimize dental implant failures and complications, biosimilar integration of dental implants to the surrounding tissue especially at the transmucosal (soft tissue) level is essential.^11^ Previously, our research group has established an effective platelet-induced functional sealing with epithelial BL attachment to the titanium surface via a protease activated receptor 4-activating peptide coating as well as identifying a novel peptide that increased epithelial cell adhesion efficacy.^12–14^ We have also reported the successful establishment of a robust perpendicular Col-I protrusions on TiO_2_ nanotube (NT) surface that impeded epithelial downgrowth.^15^

TiO_2_ NT created by electrochemical anodization has become increasingly popular due to its simplicity and low cost.^16^ Many investigators have demonstrated the potential of TiO_2_ NT to direct osteogenic differentiation of mesenchymal stem cells and promote osteoblast cell growth.^17^ The principal fibers of the periodontium are composed mainly of type-I collagen (Col-I), with densely packed bundles of Col-I fibrils oriented perpendicular to the root surface.^6,18^ An appealing feature of Col-I is the ability of Col-I monomers to self-assemble into fibrillar structures both in vivo and in vitro.^19^ Our previous achievement was to induce negatively charged triplet C-terminus of Col-I into anodic NT that supported the perpendicular protrusion Col-I.^20^ The present study had two objectives. First was to validate the integration of fibroblast-derived endogenous CoI-I to the engineered perpendicular CoI-I priming sites. In addition to the potential priming ability, Col-I has another attractive effect in tissue repair; it activates platelets via its integrin α2β1-binding domain.^21–24^ The second aim was to evaluate whether the engineered CoI-I promoted fibroblasts via platelet activation to migrate to the titanium and induce perpendicular elongation of CoI-I.

## Results

### Surface topography and wettability

**Fig. 1:**
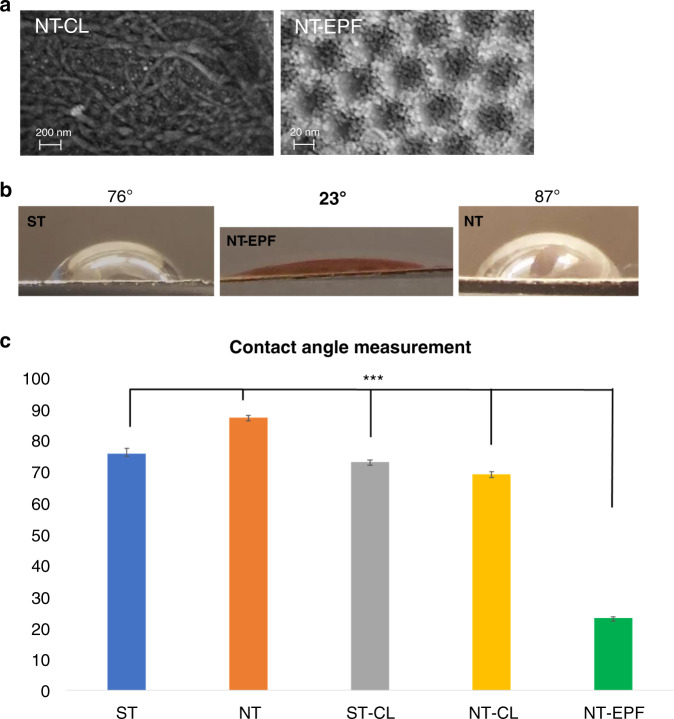
Surface fabrication and characterization. **a** SEM images of NT-CL and NT-EPF surfaces, original magnification ×100 000. Adapted with permission from previous published study by our group.^15^ **b** Representative images of water contact angle on different surfaces. **c** Average contact angle measurements

The two-step fabrication process via anodization and subsequent Col-I electrophoretic fusion (EPF) resulted in perpendicular Col-I protrusions from TiO_2_ nanotube (NT) (Fig. 1a). Scanning electron microscopy (SEM) images showed a uniform coating of collagen monomers deposited into and around the nanotubes. On the other hand, collagen fibrils lay horizontally on the NT surface, whereby Col-I was deposited through chemical-linking (CL) method. The mean contact angles were 75.92° ± 1.5° for smooth titanium (ST), 87.31° ± 0.8° for nanotube titanium (NT), 73.15° ± 0.6° for smooth titanium with chemical linking Col-I (ST-CL), 69.28° ± 0.8° for nanotube titanium with chemical linking Col-I (NT-CL), and 23.25° ± 0.4° for nanotube titanium with electrophoretic fusion of Col-I (NT-EPF) (Fig. 1b, c). The contact angle was lower in NT-EPF compared to all other surfaces (*P* < 0.000 1), indicating that the NT-EPF surface had a high surface energy and was highly hydrophilic.

### Early platelet activation, aggregation, and increased growth factor releases

**Fig. 2:**
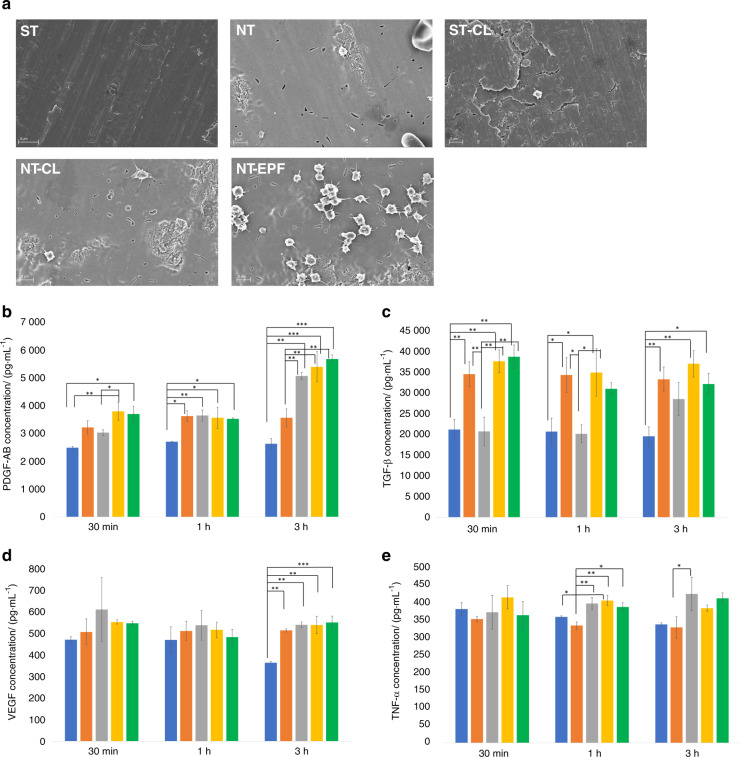
Platelet activation and growth factor releases. **a** SEM images of samples after 30-min platelet-rich plasma (PRP) incubation. **b** At 3 h, all collagen-treated surfaces (CL and EPF) have significant increase in PDGF-AB concentration. **c** At 30 min, sample surfaces with nanotube topography significantly contributed to increase in the TGF-β level. **d** At 3 h, surface treatment (both topography and Col-I coating) had an increased amount of VEGF release. **e** Col-I coating had an effect on the TNF-α concentration at 1 h (blue: ST; orange: NT; gray: ST-CL; yellow: NT-CL; green: NT-EPF) (*P < 0.05; **P < 0.01; ***P < 0.000 1)

NT-EPF surface induced platelet aggregation and activated morphology as early as 30 min while little or no attachment of platelets was seen on the other surfaces (Fig. 2a). All surfaces with Col-I coating, regardless of surface topography or binding methods, had a significant increase of platelet-derived growth factor-AB (PDGF-AB) concentration from 30 min to 3 h when compared to non-coated surfaces. Among all Col-I coatings, NT-EPF surface showed the highest PDGF-AB concentration at 3 h (Fig. 2b). All NT surfaces, regardless of the presence of Col-I coatings, had a significant early effect on transforming growth factor-β (TGF-β) release (Fig. 2c). Vascular endothelial growth factor (VEGF) concentrations showed a similar trend to that of PDGF-AB, with all Col-I-coated surfaces eliciting a significantly higher release at 3 h (Fig. 2d). The NT-EPF surface induced a marginal and non-significant increase in proinflammatory tumor necrosis factor-α (TNF-α) release (Fig. 2e) while the stimulations in tissue regeneration factors such as PDGF-AB, TGF-β, and VEGF were significant.

### Enhanced fibroblasts attachment

**Fig. 3:**
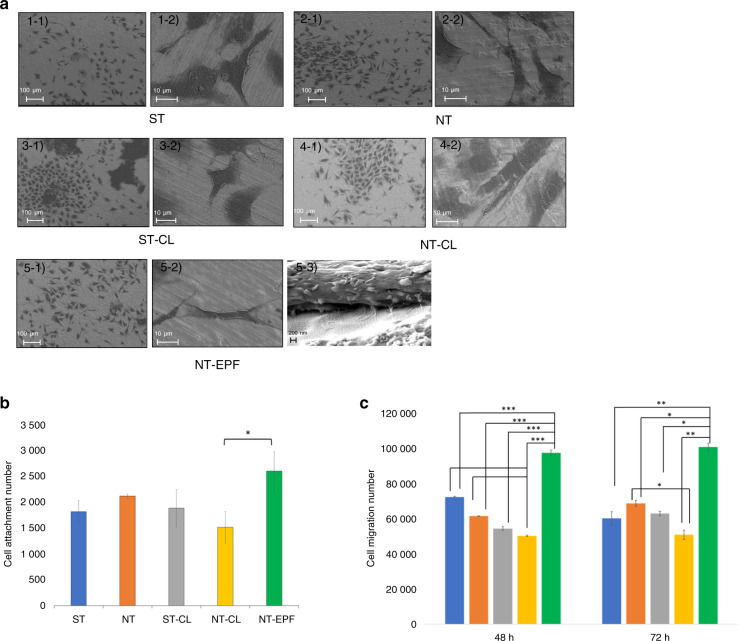
Fibroblast attachment and migration. **a** SEM images of fibroblasts after 24 h of incubation. **b** Number of fibroblasts in each group at 24 h (*p<0.05). **c** Cell migration toward various experimental surfaces at 48 and 72 h (blue: ST; orange: NT; gray: ST-CL; yellow: NT-CL; green: NT-EPF) (*P < 0.05; **P < 0.01; ***P < 0.000 1)

The mode of fibroblast attachment on each treated titanium surface was identified by SEM ultrastructure (Fig. 3a). On NT surfaces with and without Col-I coatings, fibroblasts demonstrated a more elongated spindle-cell morphology compared to the more rounded shapes on ST surfaces, with NT-EPF showing filopodia extension. The SEM image taken at a 30° tilted angle (Fig. 3a, 5–3) further demonstrated the direct attachment between the filopodia and the surface. Quantification of attached number of fibroblasts with a cell luminescent viability kit on each treated surface revealed a significantly higher number of fibroblasts on NT-EPF than on NT-CL (Fig. 3b).

### Platelet-induced fibroblasts migration

For the “Down” assay model, we observed that the NT-EPF surface significantly attracted more fibroblasts to migrate toward the surface at both 48 h and 72 h (Fig. 3c). Interestingly, the Col-I coating with the CL method did not have the same effect on fibroblast migration; in fact, the NT-CL surface had the least number of viable cells at 72 h.

### Perpendicular collagen fibril extension from perpendicular Col-engineered surface

**Fig. 4:**
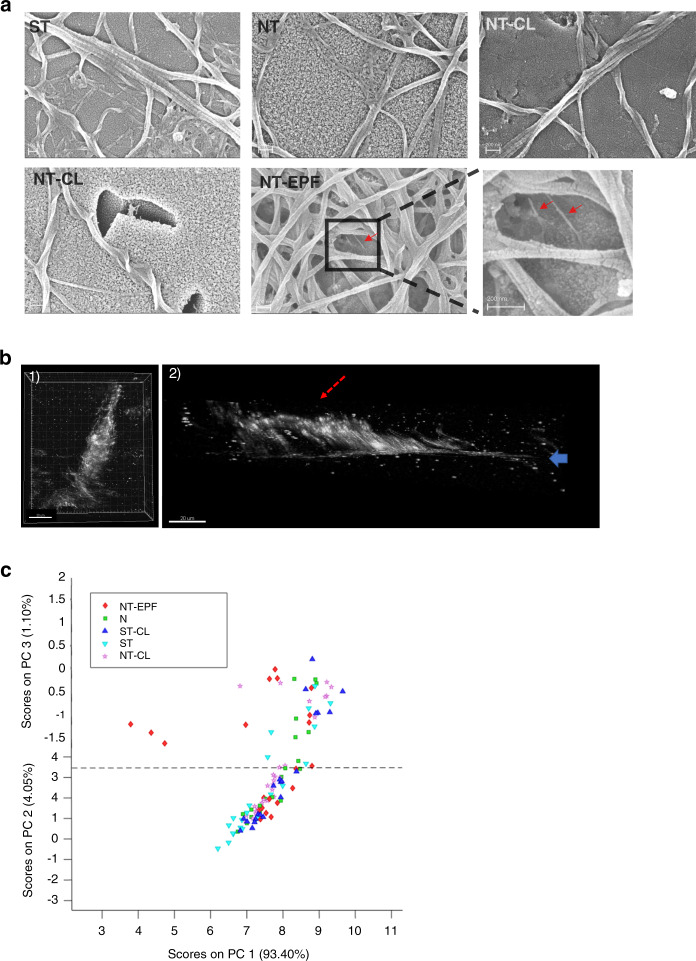
Perpendicular collagen fibril extension. **a** SEM images of fibroblast-derived Col-I deposition on various sample surfaces. Note higher magnification of NT-EPF surface showing smaller fibrillar structures (red arrows) with direct insertion to the NT-EPF surface. **b** Second-harmonic generation (SHG) microscopy images. (1) Top view of 3D reconstructed image collagen deposition on NT-EPF surface. (2) Cross-sectional view captures collagen fibrils (red arrow) inserting obliquely to the NT-EPF surface (blue arrow). **c** Principal component analysis (PCA) of Raman spectrums in the fingerprint range (700–1 800 cm^−1^)

After 3 days of incubation, greater number of collagen fibrillar bundles were seen on NT-EPF surface (Fig. 4a). While larger-sized fibrillar bundles lay flat on the surface, small fibrils rose directly from the NT-EPF surface suggesting initial perpendicular priming of each bundle (Fig. 4a). To visualize collagen in its native state without dehydration nor labeling, second-harmonic generation (SHG) microscopy was utilized. Three-dimensional reconstruction and cross-sectional views of SHG images of the collagen deposited on NT-EPF surface demonstrated direct attachment in an oblique fashion (Fig. 4b).

The unique orientation of the collagen extensions on the NT-EPF surface compared to others was further confirmed by two confocal Raman spectroscopy (CRS) spectral analyses. After applying principal component analysis (PCA) to spectral data in the fingerprint range (700–1800 cm^−1^), we found that the NT-EPF surface data deviated from all other surfaces (Fig. 4c).

Furthermore, when the spectral data were fitted to sinusoidal curves, it was obvious that the NT-EPF surface fitting curve was different from all other surfaces. Since the amplitude of the fitting curve was directly related to the anisotropic response of the amide I band, a one-way ANOVA was done for the pr1 × pr2 parameter and it was found that the amplitude of the fitting curve for NT-EPF was significantly lower than other surfaces (*P* < 0.000 1). The endogenous collagen assembly detected at the NT-EPF metal surface level had an orientation that differed from all others and indicated direct attachment to the titanium metal surface in a perpendicular/oblique fashion.

## Discussion

Most studies investigating the different surface characteristics on mucosal barrier formation did not observe differences among different implant systems^25^ nor did surface characteristics (i.e. rough sandblasted, fine sandblasted, and polished) influence the healing pattern of the peri-implant mucosal tissues.^26^ In contrast, studies by Nevins et al.^8,27^ suggested that it may be possible to promote the formation of perpendicularly attached CT fibers to the implant surface. In these studies, a polarized light microscopy revealed functionally oriented collagen fibers attaching perpendicularly to precisely defined microchannels (8–12 µm in width and 6–12 µm in depth) created by laser ablation. It has been suggested that by altering the chemical characteristic of the implant surface rather than its topography, a perpendicular orientation of CT attachment can be achieved. A mixture of parallel and perpendicular CT orientation has been observed at chemically modified hydrophilic implant abutment.^28^

In the present study, we combined both surface topography modification as well as chemical bioactive molecular coating to facilitate a soft tissue seal. It has been shown that diameters of smaller than 100 nm are optimal for enhanced cellular adhesion due to increased surface energy.^29,30^ The dental implant abutment is a transmucosal device that interacts with both the keratinocytes and fibroblasts. A previous study has shown that epithelial cells and fibroblasts respond differently to nanoscale topography, with increased dermal fibroblast and decrease epidermal keratinocyte adhesion, proliferation, and differentiation on NT arrays with a diameter of 70–90 nm and a length of 1–1.5 µm.^31^ Our previous published data with epithelial cells as well as current result with fibroblasts attachment assay somewhat corroborate previous research.^15^ Although no differences in fibroblasts cell numbers were observed at the 3-h mark, our result showed a statistically significant higher number of fibroblasts on the NT-EPF surface in the 24-h fibroblasts attachment assay. This was likely due to the high surface hydrophilicity. Regarding the morphology, more spindle-shaped cells were observed on all the NT surfaces. As demonstrated in the high magnification (5 x 10^4^) SEM image taken at 30° tilted angle, the fibroblasts cell attached tightly to the nanotubes and extended fibrillar processes to the surface, suggesting focal adhesion kinase dependent migration and cell growth.^32^ Our findings are in agreement with previous studies showing a positive influence of nano-topography on fibroblasts behavior.^33^ Furthermore, the behavior of the fibroblasts on the NT-EPF surface resembled what was observed on Laser-lok surface morphologically which was shown to induce a direct CT attachment in a human histological studies.^8,27,34^

After implantation of a biomaterial, blood–implant contact heralds the biological evens that occurs in the wound-healing process. Therefore, early response of blood platelets to the titanium surface would significantly influence subsequent biological healing through modulation of early tissue healing microenvironments via the formation of a temporary fibrin matrix scaffolds and release of growth factors and cytokines. Our SEM results showed that the NT-EPF surface was able to induce platelet adhesion and activation at 30 min when there were few platelets presents on other surfaces. Interestingly, in one study examining the effect of platelet adhesion on titanium oxide, a thinner oxide layer obtained by H_2_O_2_ positively influenced the number of adhered platelets while a thick oxide layer formed by heat treatment had a negative influence.^35^ This may explain the reason why our NT-EPF surface showed an immediate effect on platelets. The post-anodization treatment with H_2_O_2_ and subsequent Col-I binding with the EPF method rendered the surface hydrophilic.

A study published by Park et al.^36^ showed that nano-topography positively modulated the immediate blood platelet function and early macrophage immunoinflammatory response. Our results indicated that the nano-topography has a marked effect on the release of TGF-β and a slight stimulatory effect on PDGF-AB, which was masked by the greater influence of Col-I coatings. Since Col-I is a known platelet activator, all Col-I treated surface, whether through CL or EPF, upregulated the release of PDGF-AB and VEGF.^37^ Both PDGF-AB and TGF-β are well known for their positive roles in fibroblasts migration and collagen synthesis.^38–40^ In our “Down” culture model assay, we have shown that the combination of surface topography, chemistry, and subsequent activation of platelet had a positive effect on the migration of fibroblasts.

In the cell-insert culture model, we simulated the in vivo condition whereby fibroblasts secreted proteins physiosorbed onto the titanium surface. At the end of the incubation period at 3 days, we found that an abundant amount of collagen fibrils had deposited on the NT-EPF surface, forming a crisscross pattern (Fig. 4a). On the contrary, collagen fibrils were dispersed more sparsely on the other surfaces (Fig. 4a). Previous studies have investigated the relationship between different surface characteristics to the amount of protein absorption as it correlated to cell attachment and cell spreading.^41,42^ The ST and NT surfaces, both with a contact angle between 75° and 90°, did not favor collagen absorption. Interestingly, collagen fibrils were also scarce on ST-CL and NT-CL surfaces which were pre-coated with collagen; we speculate that this may be due to the instability and susceptibility to degradation of the pre-coated horizontal collagen by collagenases in the culture system. Another speculation is that the parallel collagen sheet deposited by the CL method does not offer free collagen terminus for cross-linking while the perpendicular Col-I on the NT-EPF surface does.

A notable observation obtained from the SEM picture of NT-EPF samples (Fig. 4a) after 3-day co-culture was the presence of small fibrils that appeared to attach directly to the surface in a perpendicular fashion underneath a largely horizontally laid larger fibrillar network. This was observed on multiple areas on all NT-EPF samples but not on any of the other sample surfaces. This indicated that initial collagen fibril extension were indeed perpendicular; however, we could not rule out the possibility that larger bundles could similarly extend in a perpendicular fashion because the dehydration and sputtering process required for SEM sample preparation would compress the larger bundles and thus made them lie flat on the surfaces.

To overcome the limitations of SEM, we sought for tools that allowed for in situ analysis of the sample surfaces in phosphate-buffered saline (PBS) medium. To our knowledge, we are the first to employ SHG microscopy and Raman spectroscopy to investigate the interface between cell-secreted collagen-rich extracellular matrix and titanium surface. Both modalities have the advantage of minimal sample preparations so that the delicate interface between cell-secreted collagen and the titanium is not disturbed. SHG takes place when the electric field of the exciting light is sufficiently strong to deform a molecule. If the molecule is not symmetrical, the resulting anisotropy creates an oscillating field at twice the frequency, the second harmonic. Collagen is the strongest source of second harmonics in animal tissue due to its unusual molecular structure and its high degree of crystallinity. As clearly seen in Fig. 4b, collagen could be detected by SHG with clear orientation information in the *xy* plane. Unfortunately, the technique is not without its limitation. It has been pointed out that collagen fibers with smaller diameters could not be detected by SHG.^43^

Polarized Raman spectroscopy is a vibrational spectroscopy technique that can provide information regarding chemical composition in materials. It is based on the analysis of the inelastic scattering of light interacting with molecules in which frequency shift between the incident and the scattered light is associated with a particular vibration mode of a chemical bond. Confocal Raman microscopy is non-invasive and provides chemical information with high spatial resolution. The amide I band, mainly due to C = O stretching is highly anisotropic and thus has been selected to perform composition analysis in biological tissue such as bone.^44^ Following the method proposed by Masic and others,^45^^,46^ we were able to detect differences in the anisotropic response of the Amid I band at the NT-EPF surface which suggested that collagen fiber attached to the NT-EPF surface in a non-parallel fashion.

### Conclusion

The nanotube surface topography combining with the unique perpendicular Col-I coating achieved via EPF worked in concert to promote effective fibroblasts attachment and rapid platelet activation. Furthermore, the surface stimulated increased collagen deposition by fibroblasts and the deposited collagen fibrils seemed to retain a perpendicular direction. Within the limitation of this study, we could conclude that the surface has great potential toward reducing complications and failure of dental implants by the establishment of a strong soft tissue seal. Future studies with an in vivo model are needed to further assess the quality of soft tissue attachment and clinical effects of the nanotube with perpendicular collagen protrusion surface.

## Materials and methods

### Modification of titanium surface

**Fig. 5:**
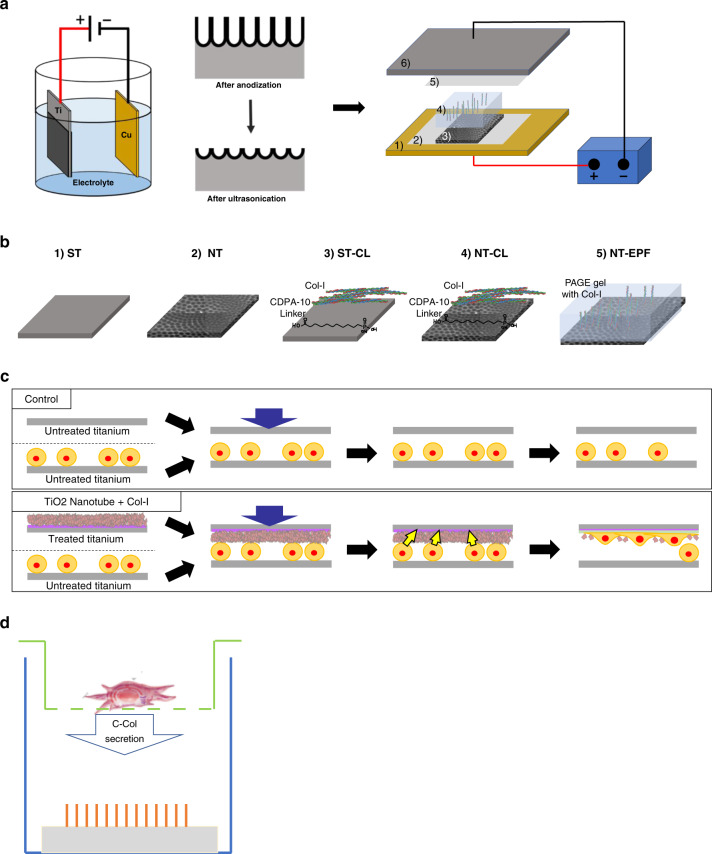
Experiment set-up. **a** Schematic representation of TiO_2_ nanotubes fabrication and Col-I electrophoretic fusion (EPF). Adapted with permission from previous published study by our group.^15^ **b** Schematic representation of specimen groups. (1) Smooth titanium (ST); (2) nanotube titanium (NT); (3) smooth titanium with chemical linking Col-I (ST-CL); (4) nanotube titanium with chemical linking Col-I (NT-CL); and (5) nanotube titanium with electrophoretic fusion of Col-I (NT-EPF). **c** Schematic representation of the down culture model. **d** Schematic representation of fibroblast culture insert model

For the present study, smooth titanium (Ti) plate with 0.1 mm thickness (Gallium Source, LLC, CA, US) was used. The surface modification was performed in two steps according to our previously published protocol.^15^ First, the nanotube (NT) array was fabricated on the titanium surface by anodization, with copper as the cathode (Fig. 5a left). Before anodization, titanium and copper samples were ultrasonically cleaned in 0.5% sodium dodecyl sulfate (SDS; Sigma, MO, USA), deionized water, acetone (Sigma, MO, USA), and ethanol (Sigma, MO, USA), sequentially, for 20 min in each solvent, and then air-dried. The distance between the anodic titanium and cathodic copper was 2.5 cm. The anodization was performed in an electrolyte solution of ammonium fluoride (NH_4_F) at 0.38 wt% and H_2_O at 1.79 wt% in ethylene glycol with constant voltages of 30 V for 3 h. Specimens were washed in two-step sonication with 30% H_2_O_2_ for 5 min first, and then in 0.1 M acetic acid for 60 min.

Second, to immobilize Col-I (Atelocollagen: Atelo Cell^®^ IPC-30, KOKEN, Japan) into the NT array in a perpendicular fashion, a new approach dubbed EPF was carried out in the semi-dry transfer system (Fig. 5a, right). The transfer unit was assembled in the following order from the bottom: (1) anode of a semi-dry blotter (Trans-Blot Turbo Transfer System®, Bio-Rad, CA, USA); (2) 1× Tris glycine buffer (TGB)-wetted filter paper; (3) titanium specimens: smooth Ti (ST) and nanotube Ti (NT); (4) 10% Native PAGE gel (Bio-Rad, CA, USA) with 10% Col-I solution; (5) 1× TGB-wetted filter paper; and (6) cathode. The 10% native PAGE gel was casted according to the manufacturer’s instructions. The Col-I solution (3 mg·mL^−1^) was mixed into the gel at a 1:10 ratio to achieve a final concentration of 0.3 mg·mL^−1^. After the polymerization reaction was completed at room temperature for 20 min, the gel was stored at 4 °C overnight prior to use. Col-I was run at a constant voltage of 25 V for 3 min. After the transfer, the specimens were washed three times with PBS to remove unwanted residues.

In total, five types of sample surfaces were prepared (Fig. 5b): (1) ST; (2) NT; (3) ST-CL; (4) NT-CL; and (5) NT-EPF. For the CL method, Col-I was linked to the ST surface and NT surface (NT) by means of phosphonic acid-based chemical linker with amide-end (10-CDPA; Dojindo Molecular Technology) as reported by Sugawara el al.^12^ Briefly, 1 nM 10-CDPA was chemically bound to TiO_2_, and subsequently Col-I was coupled to the amide-end by the EDC/NHS chemistry.

### Surface characterization

Surface topography and chemistry of the TiO_2_ nanotubes with and without different Col-I immobilization were previously characterized with SEM (Zeiss Supra 55VP field emission scanning electron microscope; ZEISS, Oberkochen, Germany), an atomic force electron microscopy (Cypher AFM, Asylum Research, CA, USA), and Fourier Transform Infrared Spectroscopy in Attenuated Total Reflection mode (FTIR-ATR) (Lumos FTIR Microscope, Bruker, MA, USA).^15^ In the present study, the surface wettability was further determined by contact angle measurement with a home-built system (Center for Nanoscale System at Harvard University, MA, USA). A digital camera was used to capture an image of the 5 µL of droplet onto the six different surfaces (*n* = 3 for each surface). Subsequently, an ImageJ plug-in^47^ was used to analyze the contact angle and the mean angle of each surface was calculated.

### Platelet activation and aggregation

Fresh human whole blood collected with 10% ACD and shipped under ambient temperature (BioIVT, NY, USA) was used to prepare platelet-rich plasma (PRP) according to a previously published protocol.^48^ Tubes containing whole blood were centrifuged at 1 000 × *g* (Salvin Dental, NC, USA) for 2 min and 15 s. After the whole blood separated into two layers, the upper light-yellow plasma layer was transferred into a new tube not containing anti-coagulant. The plasma layer in the new tubes were centrifuged at the same setting for another 5 min. The plasma was separated into an upper platelet-poor plasma (PPP) layer and a red pellet consisted of concentrated platelets at the bottom. The pellet was suspended with 1 mL of PPP to make PRP. For a final PRP volume of 10 mL, 60 mL of whole blood was used. Prior to use, CaCl_2_ in PBS was added for a final concentration of 14.3 mg·mL^−1^ in order to counteract the 10% ACD.

TiO_2_ samples were placed at the bottom of a 24-well assay plate (*n* = 4 for each group). One milliliter of the PRP (1 mL per well) was inoculated onto each sample (ST, NT, ST-CL, NT-CL, and NT-EPF). The assay plate was placed on a rocking table at 37 °C for different time points (30 min, 1 h, and 3 h). At each time point, plasma was collected and stored at 4 °C until assay. In the collected plasma, levels of platelet-related growth factors and cytokines such as PDGF-AB, TGF-β, VEGF, and TNF-α were evaluated with sandwiched enzyme-linked immunosorbent assay (ELISA) kits (R&D Systems, MN, USA). The remaining samples were fixed for SEM observation (Zeiss Supra 55VP; Zeiss).

### Cell culture and maintenance of mouse fibroblast cell line (NIH3T3)

Murine fibroblasts NIH3T3 cells were maintained in DMEM medium (Thermo Fisher Scientific) supplemented with 10% FBS at 37 °C in 5% CO_2_ and 95% atmospheric air. Cells maintained between 4–10 passages were used in the following experiments.

### Cell attachment assay

The attachment of fibroblasts to the titanium surface was investigated as attached number of viable cells and morphology by SEM. Viable cell number was evaluated with a cell luminescent viability kit (CellTiter-Glo®, Promega, WI, USA). Cells were seeded on sample surfaces (ST, NT, ST-CL, NT-CL, and NT-EPF) at 1 x 10^5^ cells·cm^−^^2^ in 200 µL of culture medium in each well of 48-well plate and settled in the culture for 24, 48, and 72 h. Unbound cells were then gently washed off with the 37 °C prewarmed culture medium and the specimens were transferred to a new well where a 200 µL mixture of culture medium and CellTiterGlo solution was added at 1:1 ratio. Cell lysis was induced by 2 min of vigorous shaking with an orbital shaker and the plate was left to stabilize for 10 min before luminescent reading. Cells on each sample surface were washed with PBS and fixed in 4% paraformaldehyde (PFA) for SEM analysis (Zeiss Supra 55VP; Zeiss).

### Cell migration assay

To evaluate the effects of the combination of different surface characteristics and platelet-derived factors on fibroblasts, a down culture model was employed to see active migration and attachment to TiO_2_ (Fig. 5c). In a 48-well cell culture-treated plate, fibroblasts were seeded at 1 x 10^5^ cells·cm^−^^2^ in 400 µL of culture medium and settled overnight in the incubator at 37 °C in 5% CO_2_ and 95% atmospheric air. The next day, titanium specimens with the same five types of surfaces (*n* = 4 per each surface type) were incubated with activated PRP for 3 h as described previously. At the end of the incubation, the samples were washed with PBS and transferred into the 48-well plate now containing overnight culture of fibroblasts. The specimens were placed with the treated surface facing down to the bottom of the well. At 48 and 72 h, the specimens were gently rinsed to remove unbound cells and processed for evaluations with a cell luminescent viability kit (CellTiter-Glo®, Promega, WI, USA) and SEM (Zeiss Supra 55VP, Zeiss).

### Col-I fiber extension assay

To verify whether the engineered Col-I on the TiO_2_ surfaces will serve as the priming sites for endogenous fibroblast-derived collagen to ligate to, transwell inserts with 3-µm-mesh permeable support in a 12-well plate (Thermo Scientific, USA) was used. TiO_2_ samples (ST, NT, ST-CL, NT-CL, and NT-EPF) were placed on the bottom of a 12-well plate. NIH3T3 cells were seeded in transwell inserts to allow secreted proteins including Col-I to fall onto TiO_2_ on the bottom of assay well (Fig. 5d). Fibroblasts were seeded at 100k per 1 cm^2^ for a total working volume of 1 µL per culture insert according to the manufacturer’s protocol for 3 days. l-Ascorbic acid was supplemented at 50 mg·mL^−1^ to the cell culture to stimulate collagen production. At the end of the incubation period, the specimens were carefully rinsed with PBS and processed for SEM (Zeiss Ultra55; Zeiss), SHG with Multi-photon Laser Scanning Microscopy (Olympus Fluoview FV1200, Japan), and CRS (HORIBA LabRam Evolution, Kyoto, Japan) analysis.

For SEM, samples were fixed in 4% PFA, washed in water, and dehydrated in ethanol series to 100% (75%, 80%, 85%, 90%, 95%, and 100%, 20 min in each incubation). Prior to SEM examination, the surface was sputtered coated with 5 nm of gold.

For SHG microscopy, samples were stored in PBS without fixation. SHG imaging was obtained with a ×20 1.2 NA water-immersion objective and a tunable Mai Tai laser source tuned to an excitation wavelength of 910 nm to produce backward SHG signal at 455 nm. The images were processed with IMARIS software (Bitplane, Switzerland).

For CRS, samples were fixed with 4% PFA and stored in PBS at 4 °C until observation. Five regions of interest were selected on each sample surface. A continuous laser beam was focused down to a micrometer-sized spot on the sample through the microscope. A green laser (*λ*_ex_ = 532 nm) with 100% output was used in combination with a ×50 objective. The aperture was set at 100. The spectra were acquired using a synapse CCD detector behind an 800 mm spectrometer with a 600 gr·mm^−1^ grating. Using a polarization analyzer, regions of interest were scanned at different angles of polarization laser from 0° to 180° (30° intervals). Each Raman spectrum was collected with an acquisition time of 20 s, and two accumulations. The spectra analysis was performed in the fingerprint range (700–1 800 cm^−1^). Raman spectra were preprocessed using the LabSpec 6 Software (HORIBA, Japan) for baseline correction and to remove autofluorescence.

We applied two methods to further analyze the obtained CRS spectra. First, a PCA was used to examine spectral data taken from all scans in the fingerprint range (700–1 800 cm^−1^). Second, following methods and theory set forth by Masic et al.^45^^,^^49^ and Bergholt et al.,^50^ we utilized the anisotropic response of the Amide I peak (1 600–1 700 cm^−1^) to estimate collagen orientation. By averaging the spectrum of the Amide I (1 600–1 700 cm^−1^) peak from all scans taken at each region from 0 to 180°, we created a sinusoidal curve corresponding to Amide I peak intensity versus polarization angle of the laser. Next, we followed the method proposed by Schrof et al. to perform a non-linear least-square fitting procedure using the following equation:

$${{I}} = {\mathrm{pr1}}\left( {{{1}} + {\mathrm{pr2}}\left( {{{\cos2}}\left( {{{x}} - {\mathrm{pr3}}} \right)} \right)} \right),$$

where *I* was the amide I intensity response, *x* was the polarization angle of the laser (radians), and the absolute number of pr1 × pr2 was the amplitude of the fitting curve.^45^

### Statistical analysis

To compare the significant differences in the number of cells attached to the specimen surfaces as well as ELISA cytokines concentration, one-way analysis of variance (ANOVA) was conducted with Tukey HSD post hoc test to assess differences that were statistically significant. For Raman multivariate analysis, the PLS toolbox plug-in for MATLAB (Eigenvector Research, Inc., WA, USA) was used to conduct spectral normalization.
